# P-846. A Single-Institution Retrospective Analysis on the Administration of Beta-lactam Antibiotics Prior to Vancomycin in Bacteremic Patients

**DOI:** 10.1093/ofid/ofae631.1038

**Published:** 2025-01-29

**Authors:** John C Cravero, Collin M Telchik, Taylor Yakubik, Lewis Woods, Lauren Sisco

**Affiliations:** Baylor Scott & White Medical Center Temple, Texas, Temple, Texas; Baylor Scott & White Medical Center, Temple, Texas; Baylor Scott and White, Temple, Texas; Baylor Scott and White Medical Center- Temple, Temple, Texas; Baylor Scott & White Health - Temple, Temple, Texas

## Abstract

**Background:**

According to the 2021 IDSA Surviving Sepsis Guidelines, prompt administration of antibiotics is an essential recommendation for patients with bacteremia. Similarly to a recent study published by Amoah et al., we sought to determine if the sequence of antibiotic administration impacted 30-day mortality.

**Methods:**

We conducted a single-institution retrospective study at Baylor Scott & White Medical Center in Temple, Texas, on patients greater than 18 years of age admitted with sepsis and confirmed bacteremia from January 1^st^, 2021, to July 1^st^, 2023 who received sequential antibiotic administration with a Beta-lactam and vancomycin within the first 6 hours of admission time. Our primary endpoint was odds ratio for 30-day mortality.

**Results:**

Of 6143 patients generated from electronic search, 222 patients were included in the Beta-lactam first group and 16 were included in the vancomycin first group. Of these 238 patients, 132 patients were admitted to the ICU upon admission. The most common Beta-lactam antibiotic administered was piperacillin-tazobactam (58.40%) followed by Cefepime (25.47%), then Ceftriaxone (13.03%). For patients with monomicrobial bacteremia, the most common organisms isolated were *Escherichia coli* (25.21%), Methicillin resistant *Staphylococcus aureus* (MRSA) (13.45%), Methicillin sensitive *Staphylococcus aureus* (MSSA) (11.34%), and *Proteus mirabilis* (7.56%). The calculated odds ratio for 30-day mortality was 0.40 (95% CI 0.089-1.831), indicating a decreased occurrence of mortality within 30 days for patients who received Beta-lactam antibiotics first, though this result was not statistically significant due to limited sample size, particularly within the vancomycin first group.

**Conclusion:**

Although not statistically significant, our results suggest that prompt administration of Beta-lactam antibiotics prior to vancomycin may have mortality benefit in patients with bacteremia. Further confirmatory studies will be required to investigate this principle.

**Disclosures:**

**All Authors**: No reported disclosures

